# A Rare Case of Streptococcus equi Subspecies Zooepidemicus Bacteremia

**DOI:** 10.7759/cureus.59911

**Published:** 2024-05-08

**Authors:** Nikeith Shah, Taysir Al Janabi, Eric Lien, Jay Thimmapuram

**Affiliations:** 1 Internal Medicine, WellSpan York Hospital, York, USA; 2 Internal Medicine, Drexel University College of Medicine, Philadelphia, USA

**Keywords:** mode of transmission, gram positive bacteremia, infectious diseases, veterinary medicine, streptococcus zooepidemicus, streptococcus equi, zoonotic infectious disease

## Abstract

*Streptococcus equi* subspecies *zooepidemicus* (*S. zooepidemicus*) is a zoonotic pathogen that primarily infects horses, pigs, and dogs. Although rare, it has also been shown to infect humans who consume unpasteurized dairy food or have direct contact with horses. Here, we present a case of *S. zooepidemicus* bacteremia in a patient without a clear mode of transmission. An 86-year-old male with a past medical history of coronary artery disease, heart failure with reduced ejection fraction, complete heart block status post pacemaker, hypertension, hyperlipidemia, and type 2 diabetes mellitus presented to the Emergency Department with fever and chills. He had fevers and rigors for three days but denied weight loss, cough, sore throat, or rashes. In the Emergency Department, vital signs revealed a fever of 101.2 degrees Fahrenheit and a heart rate of 110 with other stable vital signs. The physical exam was unremarkable except for tachycardia, and laboratory work revealed no leukocytosis but elevated inflammatory markers and elevated lactate. Computed tomography of the chest, abdomen, and pelvis did not reveal any source of infection. Blood cultures grew *S. zooepidemicus* and the Infectious Diseases team was consulted, who started the patient on Penicillin G. Due to concern for pacer-lead infective endocarditis, transthoracic and transesophageal echocardiograms were performed, which did not show valvular vegetations. Repeat blood cultures showed clearance of the infection, and the patient was ultimately discharged on amoxicillin. While our patient denied consuming unpasteurized dairy products or having direct contact with horses, upon further questioning, he did endorse family members who occasionally interacted with horses. This case is valuable as it adds to the sparse literature on *S. zooepidemicus* infections specifically in humans. Extensive history taking is of utmost importance when a clear source of infection is not easily identifiable. Further research is also needed to better understand the various modes of transmission of this bacterium to better target and caution those at an increased risk of infection.

## Introduction

The bacterium *Streptococcus equi* subspecies *zooepidemicus *(*S. zooepidemicus*) is a β-hemolytic Lancefield group C streptococcus. It is a commensal organism in horses but has the potential to become pathogenic. Infections with *S. zooepidemicus* have been seen in horses, dogs, and even humans [1-4]. Infections with *S. zooepidemicus* have been seen in humans who have either consumed unpasteurized dairy products or have had direct contact with infected horses, dogs, or cats [1,5,6]. In humans, the first documented outbreak of *S. zooepidemicus* was described in 1988, with the source of infection being the consumption of unpasteurized milk products [6]. The clinical manifestations of human infections with *S. zooepidemicus* are varied. Pelkonen et al. described three cases of *S. zooepidemicus* infections among horse farm workers in Finland who developed different complications including septic arthritis, psoas abscess, meningitis, and progressive endocarditis [1]. Other reported clinical manifestations of *S. zooepidemicus* infections included pneumonia and pharyngitis, which can lead to acute post-streptococcal glomerulonephritis [7].

## Case presentation

**Figure 1:**
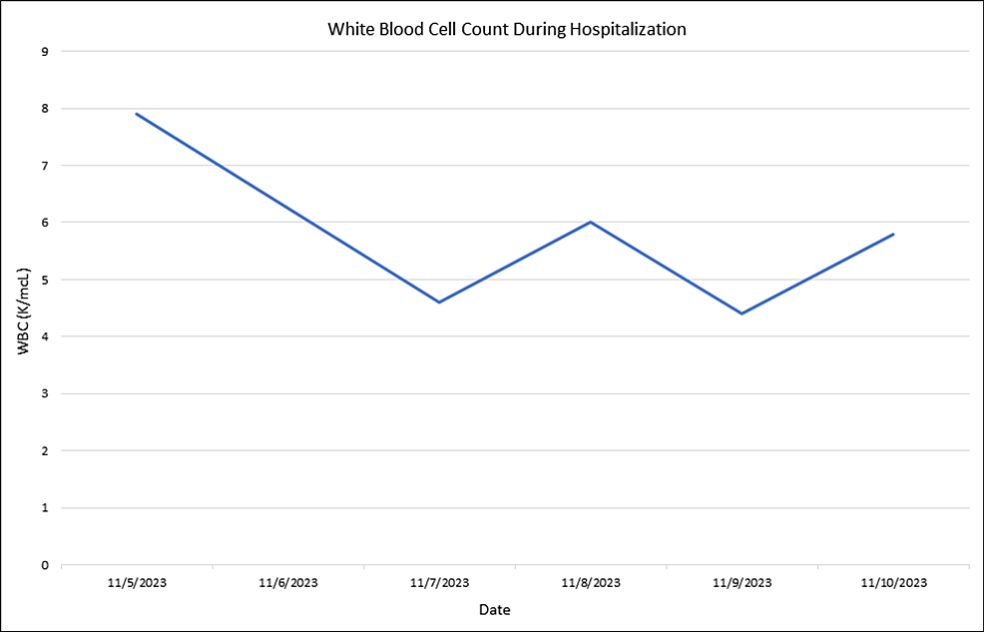
White blood cell count during hospital course

**Figure 2:**
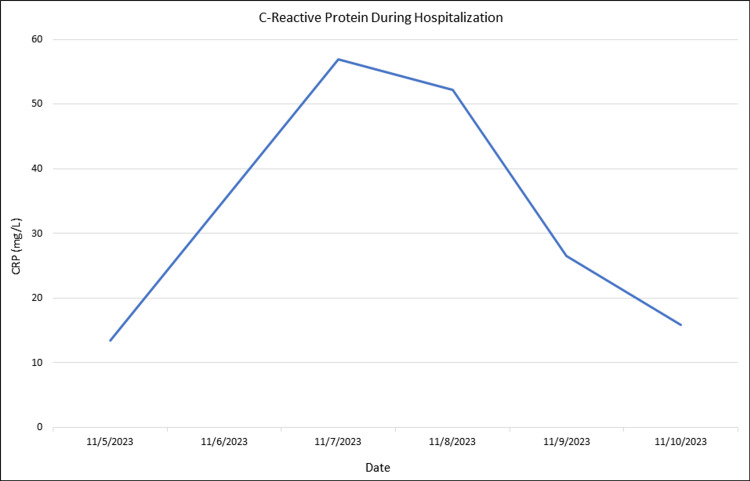
C-reactive protein levels during the hospital course

An 86-year-old male with a past medical history of coronary artery disease, heart failure with reduced ejection fraction, complete heart block with pacemaker placement, hypertension, hyperlipidemia, and uncontrolled type 2 diabetes mellitus presented to the Emergency Department (ED) with fevers and chills. His symptoms had been ongoing for three days prior to presentation, with associated rigors but no cough, sore throat, rashes, or weight loss. The patient denied any sick contacts, recent travel, or contact with sick pets or animals. In the ED, vital signs revealed a fever of 101.2 degrees Fahrenheit and a heart rate of 110 with other stable vital signs. A physical exam revealed an elderly gentleman in no acute distress with normal cardiopulmonary and neurological exam findings. Laboratory work revealed a normal white blood cell count of 4.6 K/mcL (normal range: 4.0 - 11.0 K/mcL) but an elevated C-reactive protein (CRP) of 56.9 mg/L (normal range: 0.0 - 10.0 mg/L) and lactate of 2.4 mmol/L (normal range: 0.5 - 2.0 mmol/L). His hemoglobin A1c was 8.4% (normal range: <5.7%). Computed tomography (CT) of the chest, abdomen, and pelvis did not reveal any source of infection. Blood cultures were obtained, and the patient was empirically started on vancomycin, cefepime, and metronidazole. Blood cultures later grew *S. zooepidemicus*. The Infectious Diseases (ID) team was consulted, and his antibiotic regimen was changed to Penicillin G. Given the presence of a pacemaker, there was a high concern for pacer-lead infective endocarditis. Transthoracic and transesophageal echocardiograms were therefore performed, both of which were negative for valvular vegetations. The ID team therefore thought he contracted this infection through contact with family members who had been feeding horses. During the patient's hospital course, he never developed leukocytosis, and the CRP improved over the subsequent days (Figures 1, 2). Repeat blood cultures 48 hours later showed clearance of the bacteremia, and the patient was ultimately discharged on oral amoxicillin 1,000 milligrams three times daily for 21 days, as the patient declined long-term intravenous antibiotics. After completing his antibiotic course, the patient had two surveillance blood cultures one week apart, which did not grow S. zooepidemicus. No fever, chills, diarrhea, or rash were reported afterward either.

## Discussion

The mechanism of transmission of *S. zooepidemicus* is poorly understood in our case. Most cases in the United States occurred in those who consumed unpasteurized dairy products or had direct contact with various animals, including horses, dogs, and cats. While our patient was not in contact with any animals with clear respiratory symptoms, upon further questioning, he admitted to several direct and indirect contact with animals. He was, for instance, involved in the care of his daughter’s dog on a weekly basis. His daughter would also intermittently care for her neighbor’s horse. Additionally, his granddaughter's boyfriend worked at a horse farm and frequently visited the daughter’s home. As none of the patient’s family members expressed infectious signs or symptoms, it is difficult to discern the exact host of the pathogen, considering the various environmental exposures and the nature of their family dynamics.

Another case of *S. zooepidemicus* bacteremia without a clear source of infection has been reported as well. In 2008, a 59-year-old female was hospitalized for generalized weakness and lightheadedness and was ultimately found to have *S. zooepidemicus* bacteremia with subsequent bilateral endophthalmitis, infective endocarditis, and meningitis - these were later diagnosed as secondary to septic emboli [8]. Although the patient had a horse stable on her property, she denied daily interactions with the horses or consumption of unpasteurized milk products. The patient’s husband worked with the horses on a daily basis, but neither he nor the horses were known to be sick. A dog also lived on the farm, but it was known to be healthy as well.

Another interesting aspect of our case was the treatment regimen. Our patient was treated with intravenous Penicillin G for three days followed by oral amoxicillin, as our patient did not want a peripherally inserted central catheter (PICC). Most of the cases reported in the literature thus far were treated with intravenous beta-lactam antibiotics, particularly Penicillin G, for four to six weeks. Fortunately, our patient’s treatment approach was still successful as repeat surveillance blood cultures remained negative, and the patient did not report any fevers, chills, or adverse effects related to the oral antibiotics. Our treatment approach, although different from the recommended treatment guidelines, suggests the possibility of alternative treatments being just as viable, though more research needs to be conducted [9].

## Conclusions

This case adds to the sparse literature on *S. zooepidemicus* infections, specifically those in humans. As *S. zooepidemicus* infections are much more prevalent in animals, less is known about the disease process, as it pertains to humans. When the mode of transmission of a pathogen is not clearly apparent, as evidenced in this case, extensive history-taking is very important. Even with extensive history taking, however, a clear route may still not become apparent. This case exemplifies the varied modes of transmission that are possible for pathogens. Although it appears the primary route of infection for this bacterium is via the respiratory tract, further research is needed to better understand the other routes of transmission that may be possible. Our patient was successfully treated with oral antibiotics as well, which suggests that alternative antibiotic regimens are potentially viable for the treatment of *S. zooepidemicus*.
